# RETRACTED: Scuderi et al. Efficacy of a Product Containing Xyloglucan and Pea Protein on Intestinal Barrier Function in a Partial Restraint Stress Animal Model. *Int. J. Mol. Sci.* 2022, *23*, 2269

**DOI:** 10.3390/ijms27114917

**Published:** 2026-05-29

**Authors:** Sarah Adriana Scuderi, Giovanna Casili, Marika Lanza, Alessio Ardizzone, Luca Pantaleo, Michela Campolo, Irene Paterniti, Laura Cucinotta, Salvatore Cuzzocrea, Emanuela Esposito

**Affiliations:** Department of Chemical, Biological, Pharmaceutical and Environmental Sciences, University of Messina, Viale Ferdinando Stagno D’Alcontres, 31-98166 Messina, Italy; sarahadriana.scuderi@unime.it (S.A.S.); gcasili@unime.it (G.C.); mlanza@unime.it (M.L.); alessio.ardizzone@unime.it (A.A.); campolom@unime.it (M.C.); ipaterniti@unime.it (I.P.); laura.cucinotta@unime.it (L.C.); salvator@unime.it (S.C.)

The journal retracts the article titled “Efficacy of a Product Containing Xyloglucan and Pea Protein on Intestinal Barrier Function in a Partial Restraint Stress Animal Model” [1], cited above.

Following publication, concerns were brought to the Editorial Office’s attention regarding the integrity of a number of figures presented in this article [1].

In accordance with the journal’s standard procedures, the Editorial Office and the Editorial Board conducted an investigation that identified indications of inappropriate image editing and duplication in Figures 3F and 4F, Figures 3G and 5G, and Figures 3I and 4J within [1]. Although the authors cooperated with the investigation, the explanations and supporting materials provided were not deemed sufficient by the Editorial Board to resolve the identified concerns. Consequently, the Editorial Board has lost confidence in the reliability of the reported findings and has decided to retract this publication.

This retraction was approved by the Editor-in-Chief of the journal *IJMS*.

The authors have been informed of this retraction and have indicated their disagreement with this decision.
